# Diagnosis and phylogenetic analysis of ovine pulmonary adenocarcinoma in China

**DOI:** 10.1007/s11262-013-0988-x

**Published:** 2013-10-23

**Authors:** Keshan Zhang, Hanjin Kong, Yongjie Liu, Youjun Shang, Bin Wu, Xiangtao Liu

**Affiliations:** 1 State Key Laboratory of Veterinary Etiological Biology, National Foot-and-Mouth Disease Reference Laboratory, Lanzhou Veterinary Research Institute, Chinese Academy of Agricultural Sciences, Xujiaping No.1, Yanchangpu, Lanzhou, 730046 Gansu People’s Republic of China; 2 State Key Laboratory of Agricultural Microbiology, College of Veterinary Medicine, Huazhong Agricultural University, Wuhan, 430070 Hubei People’s Republic of China

**Keywords:** China, Molecular identification, Ovine pulmonary adenocarcinoma, Phylogenetic analysis

## Abstract

Ovine pulmonary adenocarcinoma (OPA) is a lung tumor of sheep caused by jaagsiekte sheep retrovirus (JSRV). OPA is common in sheep, and it is most commonly observed in China. Without preventative vaccines and serological diagnostic tools for assay of OPA, identification of JSRV based on reverse transcription polymerase chain reaction (RT-PCR) is very important for prevention and control measures for OPA in practice management. In this study, the diagnosis of OPA was made from analysis of clinical signs, pathological observations, JSRV-like particle discovery, and RT-PCR of the target env gene. The phylogenetic analysis showed that the China Shandong (SD) strain studied in this article belonged to exogenous JSRV, and it was very similar to 92k3, which was isolated from sheep in the Kenya (Y18305). The current study reported a severe outbreak of OPA in Shandong Province, China. The observations could offer a comparative view of the env gene of JSRV.

## Introduction

Ovine pulmonary adenocarcinoma (OPA), also known as ovine pulmonary adenomatosis and jaagsiekte, is a contagious neoplasm affecting the lungs of sheep, which is caused by jaagsiekte sheep retrovirus (JSRV) [1–3]. JSRV has been described in various breeds of sheep, rarely in goats, and does not appear to infect other animals [4, 5]. OPA is believed to be the most important disease that can affect international trade as determined by the OIE (OIE Manual, 7th Edition). The disease is characterized by fluid accumulation in the lungs, which results from the production of excess secretions by tumor cells. Filtered lung fluid from infected sheep contains viral particles, and this fluid is able to manifest the disease when inoculated intratracheally into healthy sheep [6]. OPA is the most common pulmonary tumor that affects sheep and occurs in many countries around the world, with the exception of Australia, New Zealand, and Iceland. In addition, studies on OPA are an excellent model to translate such findings to human lung carcinogenesis [7].

JSRV is classified as a beta-retrovirus and resembles a simple retrovirus, since its genomic organization contains only the essential genes characteristic of retroviruses: gag, pro, pol, and *env* [8]. Enzootic nasal tumor virus (ENTV) is the causative agent of contagious ovine and caprine nasal adenocarcinoma (ONA and CNA), which has the similar genome structure and shares high homology with JSRV. As we know, there are two ONA cases in Inner Mongolia, northern China [9], and southwestern China, respectively [10]. The JSRV *env* gene encodes the N-terminal surface (SU) and C-terminal transmembrane (TM) domains of the envelope protein (*Env*), which is linked by disulfide bonds and embedded in the membrane. After *Env* cleavage by cellular furin protease, the *Env* protein binds the cell receptor during infection [11]. The molecular mechanism responsible for transformation has been investigated extensively and involves the YXXM motif and other *Env* protein domains as we have elaborated below.

There are 15–20 copies of endogenous jaagsiekte sheep retrovirus (enJSRV) are present in the genome of sheep and goats [12, 13]. All the enJSRVs have similar gene structure with exogenous JSRV (exJSRV) and ENTV. It has been reported that there are five enJSRVs that have 85–89 % sequence identity to exogenous JSRV. Regions of sequence divergence reside in the U3 region of the LTR and three regions in Gag and Env, termed variable regions, 1, 2, and 3 (VR1–3) [14]. None of the EnJSRV envs contains the YXXM motif in the CT region of the Env TM domain and is unable to induce transformation [15]. Though enJSRV env sequences are not oncogenic, it should be noted that the expressions of enJSRV proteins have been shown to block infection by exogenous JSRV [16].

There are currently no vaccines available for this virus, and consequently, disease control relies on regular flock inspections and prompt culling of suspected cases and, in the case of ewes, their offspring. During the long incubation period, the animals remain clinically healthy so that early stages of the disease with evidence of minor respiratory involvement can be easily confused with other respiratory diseases, and even by the most experienced clinicians [17]. Thus, developing a rapid and specific diagnostic assay is an urgent priority and necessary for control or even eradication of OPA. There is also no known specific antibody response following JSRV infection [18, 19]. Consequently, there is no diagnostic serological test currently available. At present, diagnosis depends on clinical and pathological investigations of OPA in combination with identification of the infectious agent by PCR targeting the JSRV specific gene [17].

Many sheep are bred in China’s Shandong Province, which are the major sheep producing region of China. In the present study, an outbreak of OPA was diagnosed based on the clinical signs of disease, pathological investigation, and RT-PCR targeted analysis of the JSRV specific gene. The full-length *env* gene of JSRV, which was identified from sheep in the Shangdong Province area of China, was cloned and DNA sequenced. This represents the phylogenetic analysis of JSRV that was located in China compared with other isolated strains from around the world.

## Materials and methods

### Biopsies sample treatment

Biopsies of the lung were fixed in 10 % buffered formalin for hematoxylin and eosin (H&E) staining and examination by light microscopy. For transmission electron microscopic (TEM) observation, biopsies were soaked in 5 % glutaraldehyde. RNA was extracted from the lungs and froth and stored at −70 °C until used for RT-PCR.

### H&E staining and TEM observation

Sections at 4 μm in thickness were obtained using a histotome, following which the tissue samples were trimmed, dehydrated, embedded in paraffin, stained with H&E, and assessed by light microscopy [20]. Histopathological changes were analyzed by optical microscopy (OM; CX41, Japan) as described previously [21, 22]. Organelle-level changes in lung lesions were determined by transmission electron microscopy (TEM; JEM-2100, Japan), and sections were prepared and observed according to the previously published methods [23, 24].

### RNA extraction and RT-PCR

Total RNA from lung tissues and froth was extracted using an RNeasy Kit (QIAGEN) according to the manufacturer’s instructions and used as a template (the RNA treated with DNase before reverse transcription) in the RT-PCR assays. Specific primers for the exogenous OPA *env* gene were designed by the premier-5 software program and synthesized in the Shanghai Sangon Biological Engineering Technology and Services Co., Ltd., China. The forward primer sequence was TTCAGCAGCCCAGCGATTT. The reverse primer sequence was AGGGAGCTTAGGTACTTGTCC. RT-PCR for the *env* gene was performed according to conventional methods. The RT-PCR products of *env* were visualized by ethidium bromide staining of 1 % agarose gels that were resolved by electrophoresis and viewed under ultraviolet light.

### Sequencing alignment and phylogenetic analysis

The RT-PCR products of the *env* gene were then cloned into the pMD-18T expression vector (Takara, Dalian, China) and transformed into *E. coli* DH5α. Sequencing was conducted using an automated DNA sequencer (model 3770; Applied Biosystems, USA). Complete sequences of the *env* gene were submitted to the NCBI GenBank database (named China SD SPA) and assigned accession numbers (KC691273). Sequence editing was performed by using the DNAstar program (http://www.dnastar.com) [25, 26]. The *env* gene sequences were collected from GenBank (http://www.ncbi.nlm.nih.gov). Multiple alignments were produced using the ClustalW program (http://www.clustal.org) [27]. A phylogenetic tree was then constructed based on the *env* gene sequences (Table 1) using the neighbor-joining procedure [28, 29] using the MEGA version 4.0 software program (http://www.megasoftware.net) [30].

**Table 1:** Detailed information of *env* genes used in this study

S. no.	Exogenous JSRVs	Country	Accession number	Host species
1	JSRV21	UK	AF105220	Sheep
2	JS7	UK	AF357971	Sheep
3	China-SD SPA	China	KC691273	Sheep
4	NM	China	JQ837489	Sheep
5	JSRV-SA	South Africa	M80216	Sheep
6	809T	UK	Y18302	Sheep
7	83RS28	USA	Y18303	Sheep
8	84RS28	USA	Y18304	Sheep
9	92k3	Kenya	Y18305	Sheep

S. no.	Endogenous JSRVs	Country	Accession number	Host species
10	enJS5F16	South Africa	AF136224	Sheep
11	enJS56A1	South Africa	AF153615	Sheep
12	enJSRV-NM	China	DQ838493	Sheep
13	enJSRV-16	USA	EF680300	Sheep
14	enJSRV-9	USA	EF680316	Sheep
15	enJSRV-4	USA	EF680317	Sheep

S. no.	ENTVs	Country	Accession number	Host species
16	ENTV-2	Spain	AY197548	Goat
17	Sheep TNO28	Spain	Y16627	Sheep

Alignment of the envelope amino acid sequences of 12 exogenous JSRVs. JSRV21, JS7, China-SD SPA, NM, JSRV-SA, 809T, 83RS28, 84RS28, 92k3 that maintain an open reading frame along the entirety of env

## Results

### Clinical signs and necropsy

Jaagsiekte outbroke in a farm containing 400 Dorper sheep for the purposes of breeding production, in the Shandong Province of China in 2010. All the sheep were not imported from other place, and they were bred there over many generations. Clinical symptoms of OPA were observed and characterized by a fever persisting for a period of time, with even foaming developing at the mouth. Respiratory signs occurred during the acute stages of the disease, which included malodorous, mucopurulent nasal discharge, frequent sneezing, increased respiratory rate, extended head, and mouth breathing. The sheep that presented with clinical symptoms of OPA were mostly more than 2-years old. Serious lung swelling, necrotizing fester stove, grayish nodules, or extensive solid tumors were also found at necropsy of the lungs. The trachea and bronchus were filled with white froth. Morbidity of this case was estimated as 3.5 % (14/400 cases), and mortality rates were estimated as approaching 100 %. In fact, 14 sheep out of 400 were showed the OPA typical clinical symptoms. Only the representative one was selected and necropsied. White froth and lung samples from the infected sheep were collected according to the Declaration of Helsinki and the Guide for the Care and Use of Laboratory Animals (Ministry of Science and Technology of China, 2006).

**Fig. 1:**
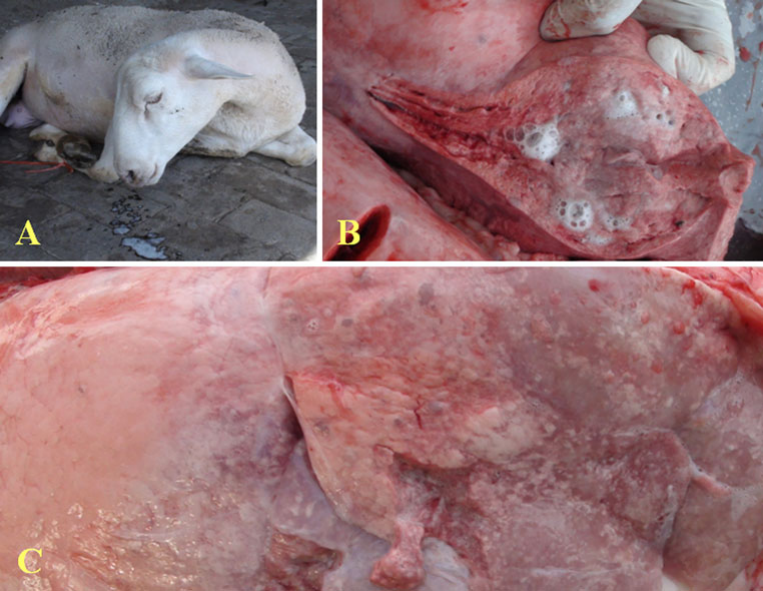
Clinical and necropsy characteristics of OPA. **a** OPA-affected animals developed progressive respiratory distress, reflected by a white mucopurulent froth nasal discharge. **b** Froth was found in the bronchus at necropsy. **c** The lungs displayed lesions characteristic of OPA, and swelling, grayish nodules, necrotizing fester stove, and extensive solid tumors (Color figure online)

Mucopurulent white froth nasal discharges of infected sheep were observed when the hind legs were lifted or drifted long time. (Fig. 1a), and were found in the bronchi at necropsy (Fig. 1b). Serious lung swellings, gray-colored nodules, and extensive solid tumors were found at necropsy in the lungs (Fig. 1c).

### Pathologic and electron microscopic findings of OPA

**Fig. 2:**
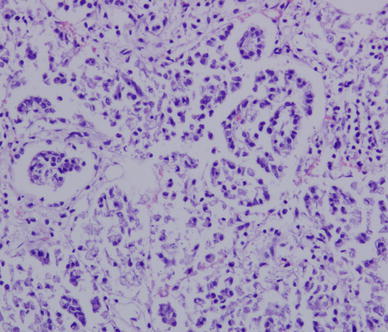
Histological observations after H&E staining of lung from infected sheep. Alveolar epithelial cell proliferation and lymphocyte infiltration in pulmonary interstitial cells were observed (H&E staining procedure, ×200 magnification)

**Fig. 3:**
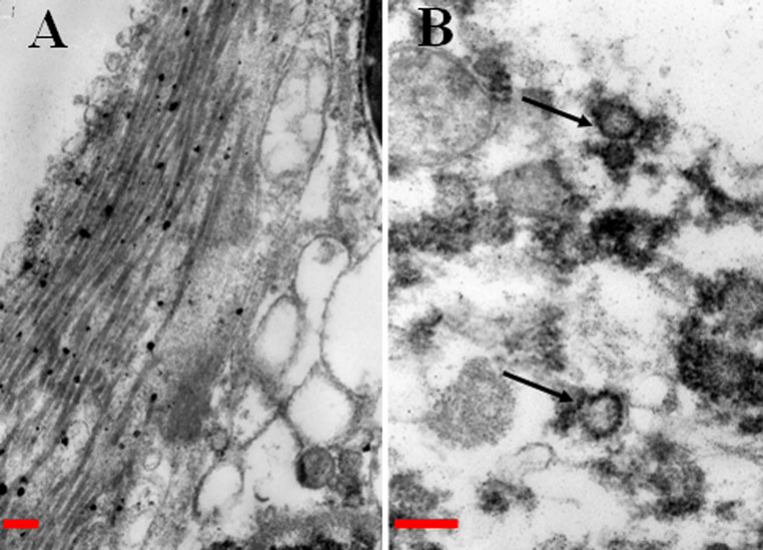
Electron microscopic observation of the lung from infected sheep. **a** Proliferation seen in collagen fibers of the alveolar mediastinum (×20,000 magnification). **b** OPA virus-like particles were found (*arrowed*), the virus diameter was found to be approximately 100–125 nm, and raised small bump-like structures were found on the surface of virus particle. The *red scale bar* represents 200 nm (Color figure online)

After H&E staining of the lungs, which were obtained from infected sheep, proliferation of alveolar epithelial cells was confirmed, which were found to burst into the alveolar cavity like the papillary. In addition, lymphocytic infiltration into the pulmonary interstitia was observed (Fig. 2). Abundant proliferative collagen fibers were also observed in the alveolar mediastinum (Fig. 3a). OPA virus-like particles were found in the lung, and the length of the virus diameter was determined to be approximately 100–125 nm, with small bump-like structures existing on the surface of the virus particles by TEM analysis (Fig. 3b).

### Gene amplification of *env* and sequencing

The expected 1,848 bp PCR fragment was visualized from total RNA extracted from infected sheep lungs, whereas no bands were observed in the negative controls. The sequencing results showed that the *env* gene had a size of 1,848 bp and consisted of 615 encoded amino acids with an average G+C ratio of approximately 40.37 % and a predicated molecular weight of 69.4 kDa. The “YXXM” motif was found in the *env* gene (aa 462–505) and located in the region of the transmembrane area (TM). The *env* gene sequences identified in this study were submitted to the NCBI GenBank and assigned appropriate accession numbers (KC691273) and named China SD SPA.

### Phylogenetic analysis

The results of sequencing alignment showed that the homology of the China SD SPA shared 93.7–96.6 % sequence identity at the nucleotide level and 95.8–99.5 % homology at the amino acid level compared with other exJSRV isolates from different regions of China. The nucleotide sequences of *env* obtained from China SD SPA shared a 96.6 % similarity with a representative strain from South Africa (Accession No. NC-001494) and a 95.0 % similarity with a representative strain from UK, JSRV21 (Accession No. AF105220). Sequence analyses of the nucleotides showed that the China SD SPA had a lower homology with endogenous *env*. For example, the China SD SPA shared only an 88.1–89.1 % similarity with endogenous *env* at the nucleotide level and a 90.7–92.5 % homology at the amino acid level. The China SD SPA strain shared the lowest homology with enJSRV, which was identified in China (DQ838494) at the deduced amino acid level. The China SD SPA strain shared a respective 89.9–89.1 % homology with Enzootic nasal tumor virus strains ENTV-2 (AY197548) obtained from goat, and TNO28 (Y16627) obtained from sheep at the amino acid level.

**Fig. 4:**
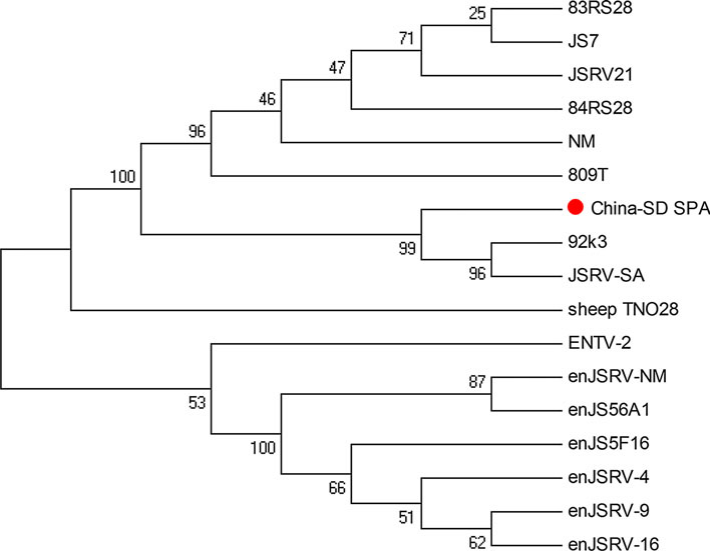
Phylogenetic analysis of different JSRV strains based on the *env* gene. The phylogenetic relationship was constructed using the neighbor-joining program of MEGA 4.0. Bootstrap analysis was performed with 1,000 trials. The *red spot* represents that the China SD SPA was the JSRV strain used in this study (Color figure online)

The phylogenetic tree data showed that the *env* genes of both exJSRV and enJSRV, which were both isolated from different origins, were divided into two different branches (Fig. 4). It showed that among exJSRV strains, the China SD SPA strain was the closest in similarity to 92k3, which was isolated from sheep in the Kenya (Y18305) (Fig. 4).

## Discussion

The methods of intensive sheep breeding are an inevitable trend, yet it provides a convenient mechanism for the spread of disease [31, 32]. OPA was first found in South Africa in 1825 [33], and it was first reported in China in 1951 [34]. Since then, OPA has been found in a variety of breeds in many countries. Not all sheep that are infected with JSRV can develop OPA [35, 36], and some breeds may be more resistant to OPA than others. OPA is an intriguing disease that presents some difficult challenges for control measures, and accounts for almost 70 % of all sheep tumors [5]. The more concerning feature of JSRV infection is that it provides conditions that favor secondary infection. OPA was previously confirmed to vertically infect from mothers to their young via the colostrum [37]. Thus, OPA is an important economic and animal welfare concern requiring comprehensive prevention and control of the disease, which is an urgent need. Shandong Province is the main sheep-breeding region of China located in the eastern coastal areas. In this study, a natural outbreak of OPA among sheep was found, and JSRV was analyzed in Shandong Province.

The typical clinical signs and macroscopic lesions of OPA were observed in infected sheep in the present study (Fig. 1a–c), which were consistent with previous reports [4, 8, 32]. Tumor characteristics were found in the lungs from infected sheep by H&E staining and observation (Figs. 2, 3a). Mycoplasma ovipneumoniae [38], Maedi-visna [39], and other pathogens can manifest similar clinical signs as those presented by OPA. For the above reasons, OPA diagnosis was based only on clinical symptoms and was thus considered to be rather inaccurate. Up to now, JSRV can replicate for a short period of time in tumor cells from young lambs [40], and ovine choroid plexus (CP) cells can be used for a permissive cell culture system for JSRV [41]. Antibody responses to JSRV have not been detected in the sera of affected sheep, even when using highly sensitive assays such as immunoblotting or enzyme-linked immunosorbent assays [19, 42]. Thus, methods of virus isolation and serological testing are not suitable for the diagnosis of OPA. To verify that JSRV existed in the lungs of infected sheep, JSRV-like particles were observed under TEM (Fig. 3b). The *env* gene was confirmed in the lungs of infected sheep by RT-PCR analysis.

To analyze the molecular features of JSRV, the *env* gene was sequenced, which showed that the *env* gene that was cloned in this study belonged to exogenous JSRV. Sequence results comparison between exJSRV and enJSRV *env* proteins indicated that TM is conserved except variable region 3 (VR3) that consists of membrane-spanning region and CT [15]. The TM area was often used in genetic evolution analyses between different strains. The domains of JSRV *Env* involved in the transmembrane have been studied extensively [43–45]. There is a tyrosine residue in the JSRV CT at position 590, while the CT of enJSRV lacks tyrosines.Y590 in the CT is important for JSRV Env transformation. The amino acid sequence surrounding Y590 is YRNM, and if Y590 is phosphorylated it could potentially bind cellular proteins with SH2 domains. YXXM and YXN are putative binding motifs for the SH2 domains of the PI3K regulatory subunit (p85) and growth factor receptor binding protein-2 (Grb-2), respectively [46]. It might be the YXXM in the JSRV CT bind to PI3K leading to downstream signaling to result in cellular transformation. Mutations of the YXXM motif can abolish or decreased transformation in cellular assays [47, 48]. One may thus conclude that the YXXM motif can be an identification criteria used to distinguish exJSRV and enJSRV. The YXXM motif that was found in the *env* gene identified from this case can help us conclude that the China SD SPA belonged to the exJSRV.

**Fig. 5:**
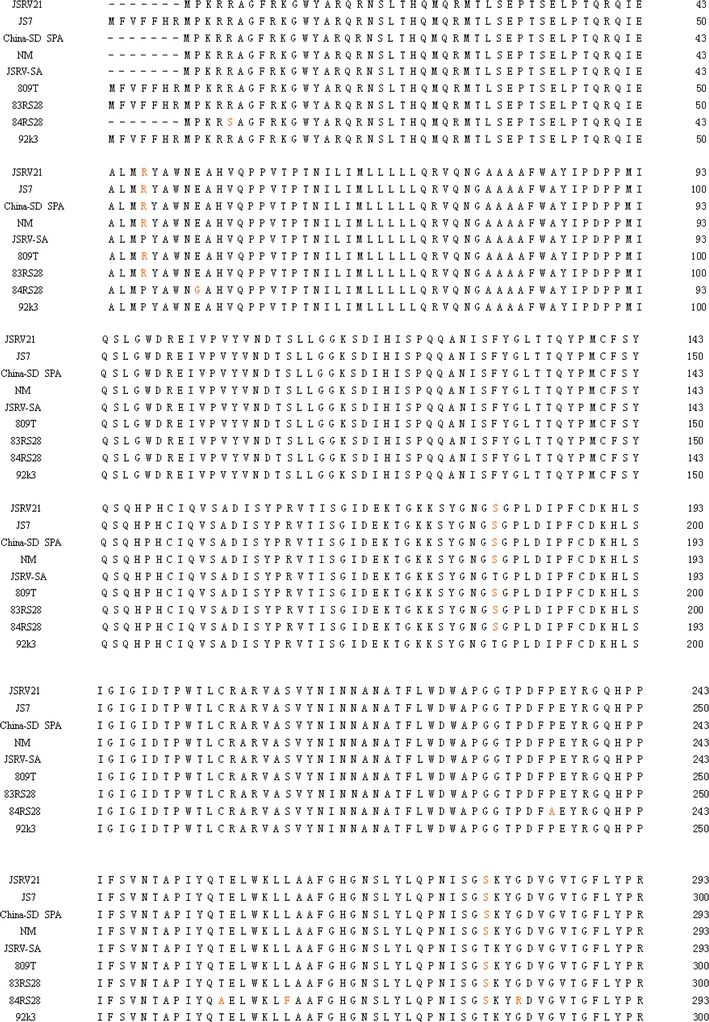

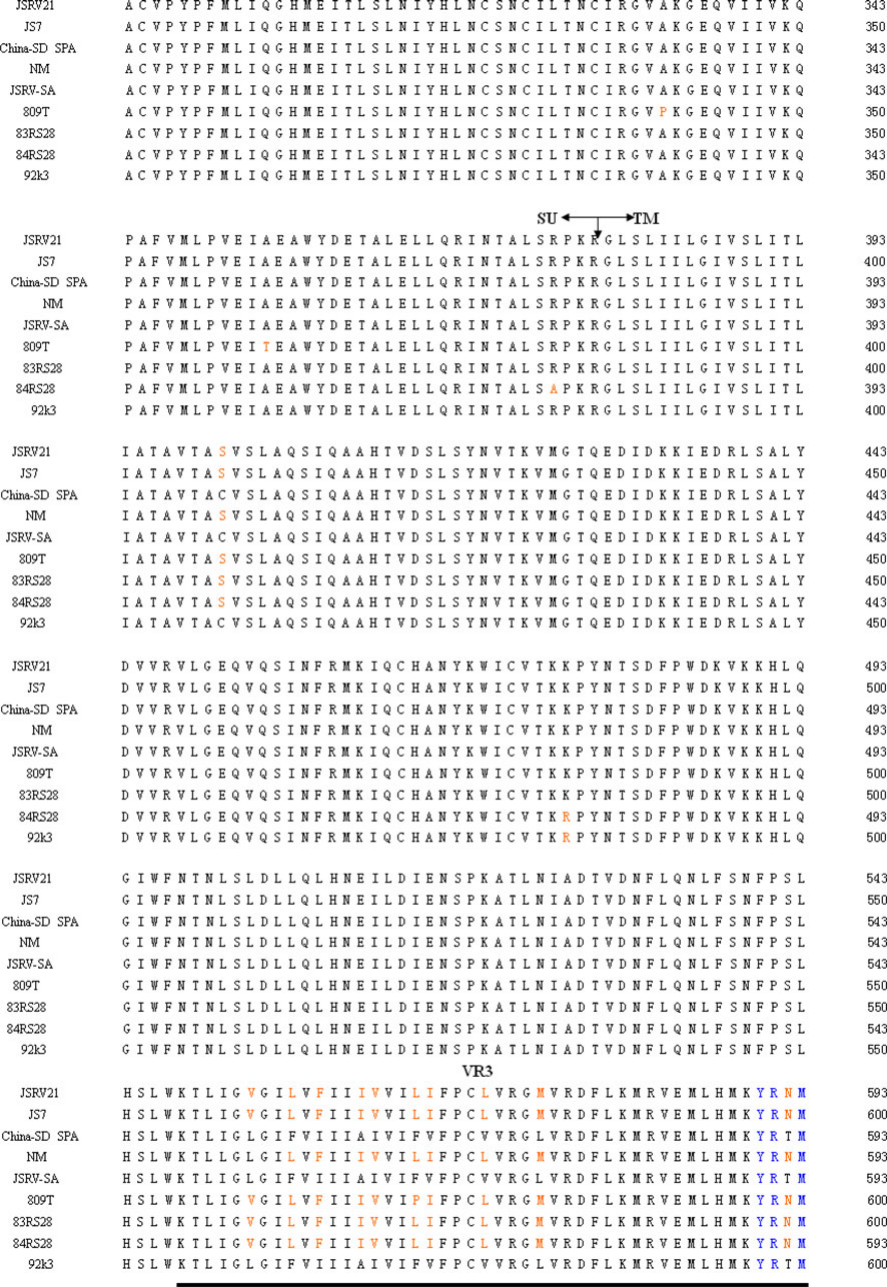

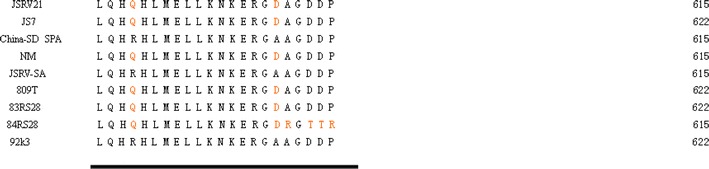
The boundary between the surface (*SU*) and transmembrane (*TM*) regions is indicated. The YXXM motif was highlighted by *blue*. VR3 is *underlined*; note the polymorphism between all sequences in VR3 (Color figure online)

Sequences of the *env* gene obtained from 21 JSRV strains were collected from GenBank (Table 1). Sequence analysis based on both amino acid and nucleotide levels indicated that the China SD SPA strain studied in this article was very close to 92k3 (Y18305). Phylogenetic analysis showed that endogenous and exogenous JSRVs belonged to different branches, respectively (Fig. 4). The exJSRV, ENTV, and enJSRV are closely related beta retroviruses with high sequence similarity. The Envelope amino acid sequences of nine exogenous JSRVs are shown (Fig. 5), and cytoplasmic tail (CT) region and YXXM motif should be highlighted, which will help us compare exact sequences among all exogenous strains derived from all over the world. This article provided phylogenetic information about a JSRV strain in China, which will be of use for prospective studies of OPA in China.
